# The Effect of Ball Burnishing on Dry Fretting

**DOI:** 10.3390/ma14227073

**Published:** 2021-11-21

**Authors:** Slawomir Swirad, Pawel Pawlus

**Affiliations:** Faculty of Mechanical Engineering and Aeronautics, Rzeszow University of Technology, Powstancow Warszawy 8 Street, 35-959 Rzeszow, Poland; s.swirad@prz.edu.pl

**Keywords:** ball burnishing, surface topography, friction, wear

## Abstract

Experiments were conducted under a dry gross fretting regime. Steel discs were put in contact with ceramic balls. Before tribological tests, discs were subjected to ball burnishing with different pressures. Due to ball burnishing, a decrease in surface amplitude and an increase in microhardness occurred. Ball burnishing caused decreases in the friction force and volumetric wear of up to 45% in comparison to sliding pairs containing milled discs. The friction force and volumetric wear were higher for a higher roughness of disc.

## 1. Introduction

The aim of ball burnishing is to improve the functional properties of the surface. During ball burnishing, the ball pressed to the machined surface moves along the assumed path. Plastic deformation occurs, without material removal. This treatment leads to a decrease in hardness, an improvement of residual stresses, and a decrease in roughness height. There are many parameters that affect surface quality after ball burnishing. Among them, burnishing speed, feed, and force are the most important [1]. Saldaña-Robles et al. [2] changed these three parameters, Jerez-Mesa et al. [3] studied the effect of burnishing speed and feed, El-Tayeb et al. [4] changed burnishing speed and force, Rodriguez [5] and Swirad and Pawlus [6] changed burnishing force. Too high a burnishing force can lead to surface deterioration. However, Rodriguez et al. [5] found that high burnishing force caused the increase in hardness even in cases of surface destruction. Typically, studies of the effects of burnishing parameters on surface roughness and hardness were performed [7,8]. Researchers tried to predict analytically the roughness of the burnished surfaces [9,10].

As the burnishing process can improve the surface quality, the effects of burnished surfaces on the improvement of functional properties were studied. These improvements are mostly connected with a hardness increase and a decrease in roughness height. Revankar et al. [11] achieved a reduction in wear and friction of more than two times due to ball burnishing of titanium alloy. Travieso-Rodriguez et al. [12] increased the fatigue lifespan of AISI 1038 specimens by up to 77% during the application of ball burnishing. Similar effects were achieved as the result of shot peening. Hardness growth caused considerable wear reduction [13,14]. El-Tayeb [4] found that burnishing led to friction reduction in comparison to the behavior of turned surfaces. Swirad and Pawlus found that ball burnishing caused reductions in wear and friction under dry sliding compared to milled samples [6]. Ball burnishing led to a decrease in the coefficient of friction in lubricated sliding [15], this effect was related to the improvement of surface quality of steel samples.

Fretting is a relative motion of low amplitude. It occurs when the sliding amplitude is smaller than the radius of the elastic contact [16]. For higher amplitude, reciprocating sliding happens. Fretting can be divided into partial slip and gross slip [17,18,19]. These regimes can be identified based on the slip index calculated on the basis of fretting loops [20,21]. Partial slip leads to fretting fatigue (cracks), while the gross slip leads to wear, typically abrasive and adhesive [22]. Fretting wear is related to created oxide debris, this effect can be positive or negative [22,23]. A decrease in the normal load caused a decrease in volumetric wear. For high contact pressure, partial slip is possible [24]. The effect of hardness on fretting wear is not clear. Oxide debris can be embedded in the softer surface, causing increased wear of a harder sample [25]. Budinski [26] found that wear increased when the difference between the hardness of two counterparts increased. Researchers obtained various effects of surface topography on fretting. An opinion exists that rougher surface debris can be accumulated in the valleys, reducing wear [27]. However, the obtained effects of surface roughness on fretting wear and friction were sometimes contradictory. Kubiak et al. [28,29] achieved a smaller coefficient of friction for rougher samples. Lenart et al. obtained various results depending on the hardness of two counterparts. For similar hardness of two steel samples, the wear volume of the tribological system was larger for higher disc roughness [30]. When a harder ball co-acted with a softer disc, the roughness height of the disc had a negligible influence on tribological properties [31]. When a steel disc contacted a ceramic ball, the smoother discs produced smaller friction and wear than the rougher discs [32]. Lu et al. [33] found that in torsional fretting wear levels were lower when the one-directional texture was perpendicular to the movement direction.

It is difficult to find in technical literature dependencies between parameters of ball burnishing and friction and wear under dry fretting. The authors of this paper try to fill this gap.

The purpose of this work is to study the effect of ball burnishing process on dry gross fretting.

## 2. Materials and Methods

Fretting tests were carried out in ball-on-disc configuration. Ceramic ball made of WC material of 10 mm diameter contacted steel disc made of 42CrMo_4_ material of 42 ± 2 HRC hardness. The experiments were carried out at 30 °C temperature, the relative humidity was 35–45%, the frequency was 50 Hz, the stroke was 0.1 mm, and the number of cycles was 45,000. The normal load changed, it obtained values of 20, 30, 40, and 50 N. For all normal loads, the elastic contact diameter was higher than the stroke. The discs had a diameter of 25.4 mm and a height of 9 mm. They were subjected to ball burnishing using the Haas CNC Vertical Mill Center VF-1 equipped with Ecoroll burnishing system (HG-6). The burnishing tool had a 6 mm diameter. Spiral burnishing strategy was chosen, burnishing speed was 500 mm/min, and burnishing width was 0.01 mm. Burnishing speed was constant during machining. The burnishing width is defined as the distance between the two next paths of the burnishing tool and was constant during machining to obtain similar surface characteristics.

There were the following burnishing pressures: 10, 20, 30, and 40 MPa. The number of test repetitions was three. During the test, the coefficient of friction was monitored.

The disc samples were milled prior to burnishing. Before tests, the measurements of disc surfaces were carried out using Talysurf CCI Lite white light interferometer (Taylor Hobson Ltd., Leicester, UK) of 0.01 nm vertical resolution. The measuring sample of 3.3 mm × 3.3 mm area contained 1024 × 1024 data points. Before the calculations of the texture parameters using TalyMap software (Gold 6.0), each surface was leveled without using digital filtration. Spikes were eliminated. Surface textures of worn samples were also studied using Phenom ProX desktop SEM (Thermo Fisher, Waltham, MA, USA).

Surface microhardness was measured using tester Reicherter Brivisor KL2 Vickers microindenters (Buehler Ltd., Lake Bluff, IL, USA) with a lens system. Tests were conducted by applying a 3 N load with a load duration of 20 s.

To obtain wear volumes, balls and discs were measured after tests. The volumetric wear of the tribological system was the sum of the wear volumes of the disc and ball [34].

**Figure 1:**
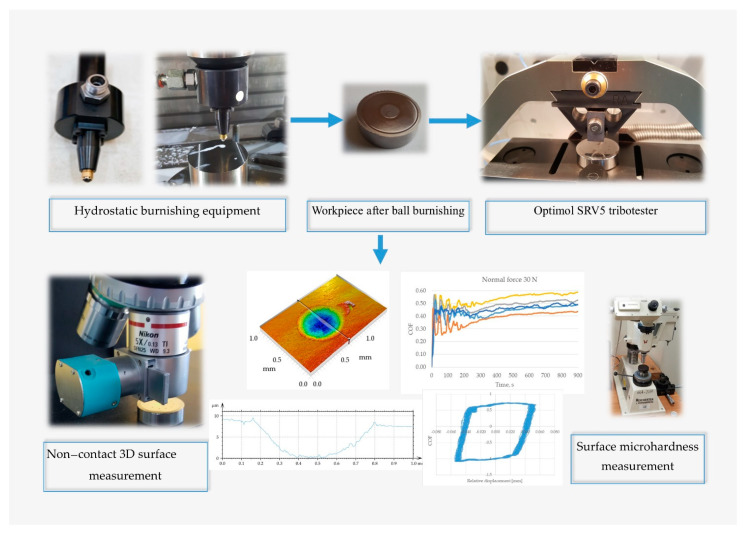
A scheme of the experimental arrangement.

Figure 1 presents a scheme of the experimental arrangement.

## 3. Results

**Figure 2:**
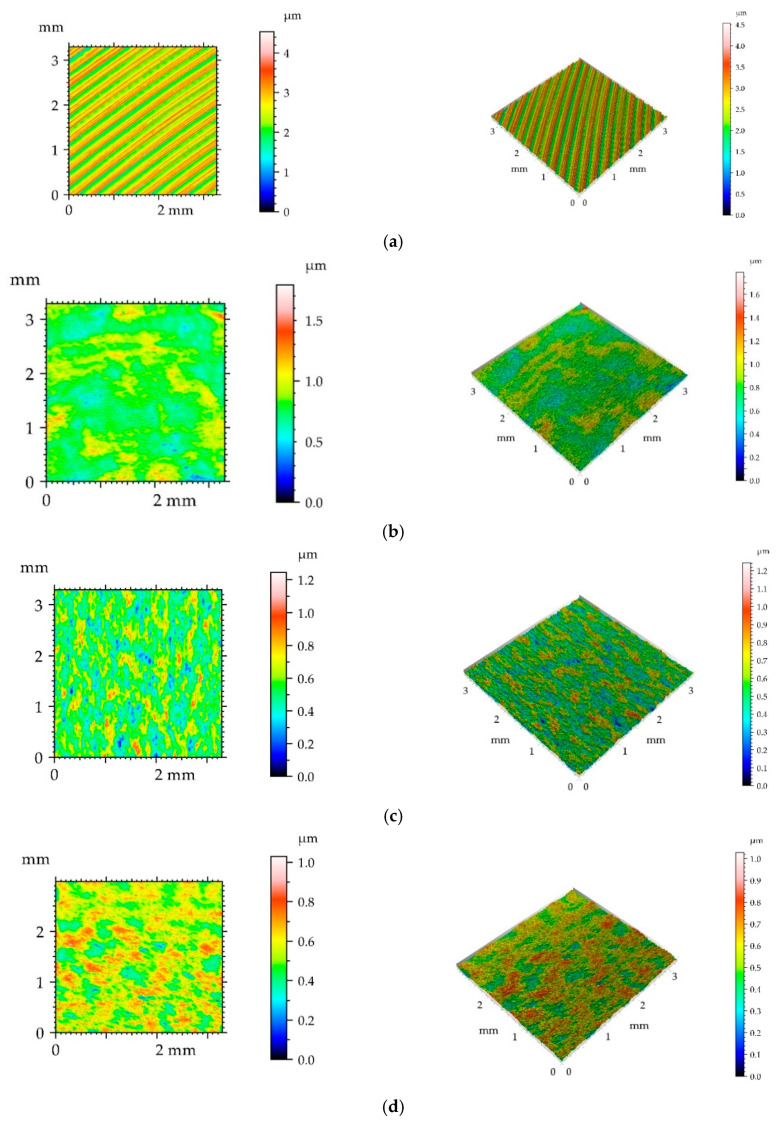

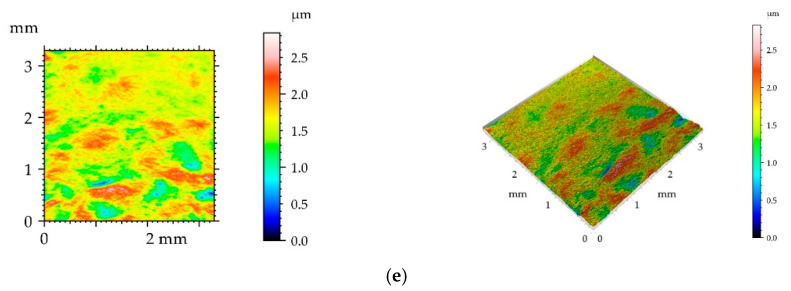
Contour plots (left) and isometric views (right) of milled disc surface (**a**), and of burnished disc surfaces with pressures of 10 (**b**), 20 (**c**), 30 (**d**), and 40 MPa (**e**).

Figure 2 presents isometric views and contour plots of machined samples.

**Figure 3:**
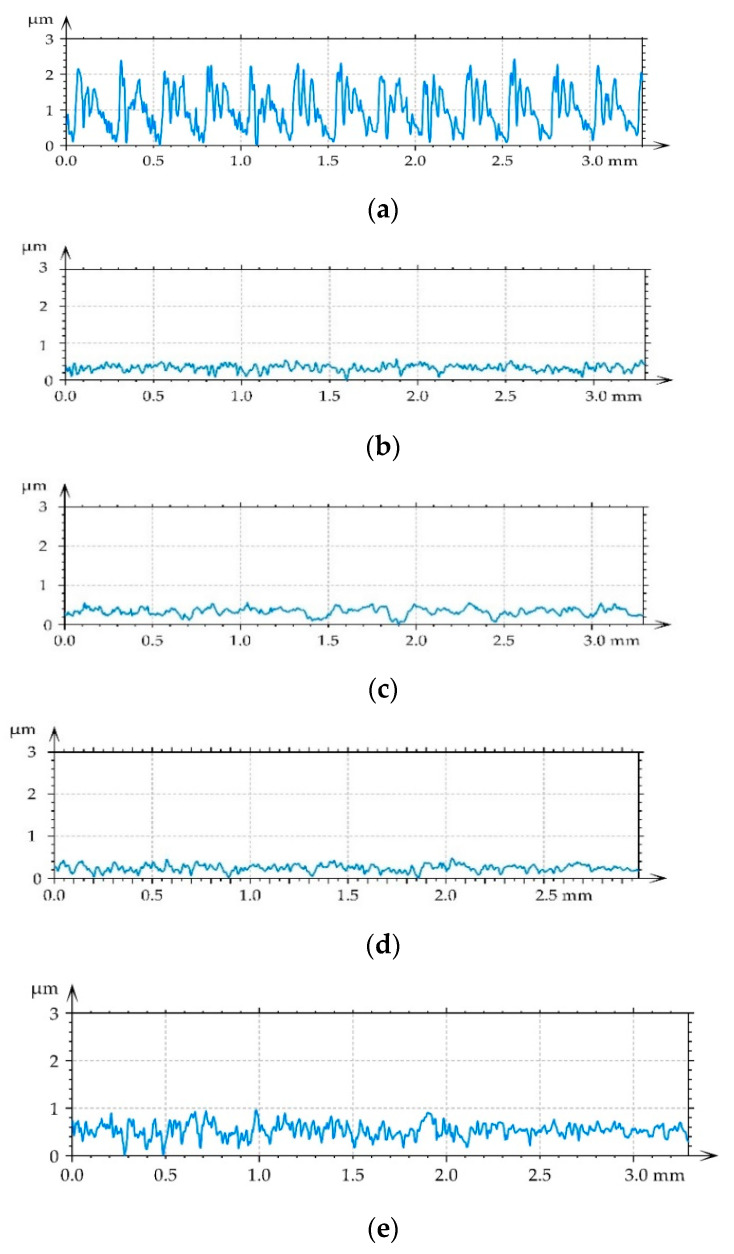
Representative roughness profiles of milled disc surface (**a**), and of burnished disc surfaces with pressures of 10 (**b**), 20 (**c**), 30 (**d**), and 40 MPa (**e**).

Figure 3 shows roughness profiles of tested disc surfaces before tests.

Table 1 lists the parameters of the disc samples according to the ISO 25172-2 standard. Reference [35] presents the definitions of these parameters.

One can see that ball burnishing caused decreases in height parameters: rms. height Sq, average height Sa, maximum height Sz, peak height Sp, valley depth Sv, and rms. slope Sdq. The decreases were the smallest for the highest pressure of 40 MPa. The correlation length Sal increased due to burnishing. The initial milled surface was one-directional anisotropic—the texture parameter Str was very small—0.014. As the results of burnishing the isotropy degree increased. The skewness Ssk decreased and kurtosis Sku increased due to burnishing. The values of these parameters confirm that the texture changed from deterministic (milled) to random (burnished). For the highest burnishing pressure surface height was not so small as those obtained for smaller pressures. Perhaps this pressure led to surface deterioration, similar behavior was found in [5,6].

**Figure 4:**
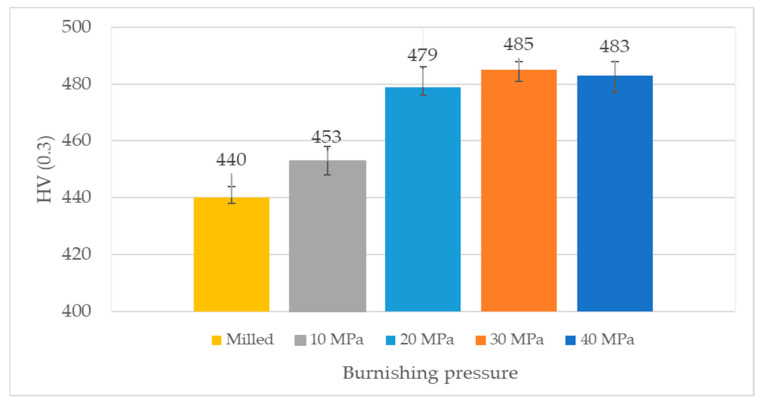
The results of microhardness measurements of discs.

Microhardness values of discs are presented in Figure 4. The five indentations were performed for each disc sample. Due to burnishing, microhardness increased. The growth was the smallest for the lowest burnishing pressure.

**Figure 5:**
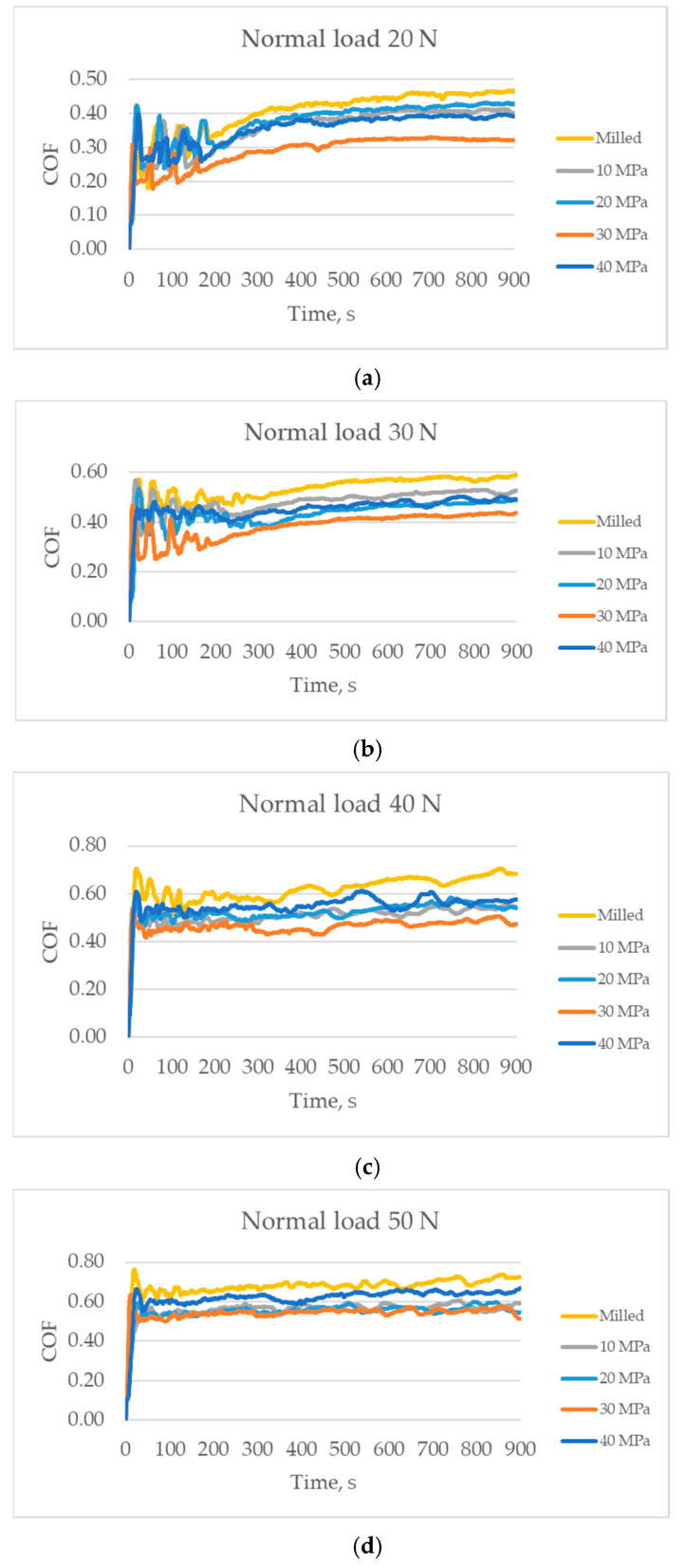
The friction coefficient as a function of time; the normal load was 20 N (**a**), 30 N (**b**), 40 N (**c**), and 50 N (**d**).

Figure 5 presents runs of the coefficient of friction versus time for various tested assemblies. For the smallest normal loads of 20 and 30 N the fluctuations of the friction force occurred for the first 200 s, from this time the friction coefficient slowly increased as the test progressed. When the normal load was 40 N, the friction force was stable after about 100 s and for the normal load of 50 N after about 50 s. For all applied loads, the highest friction was obtained for assembly with the milled surface, while the smallest was for the burnished surface with the pressure of 30 N.

**Figure 6:**
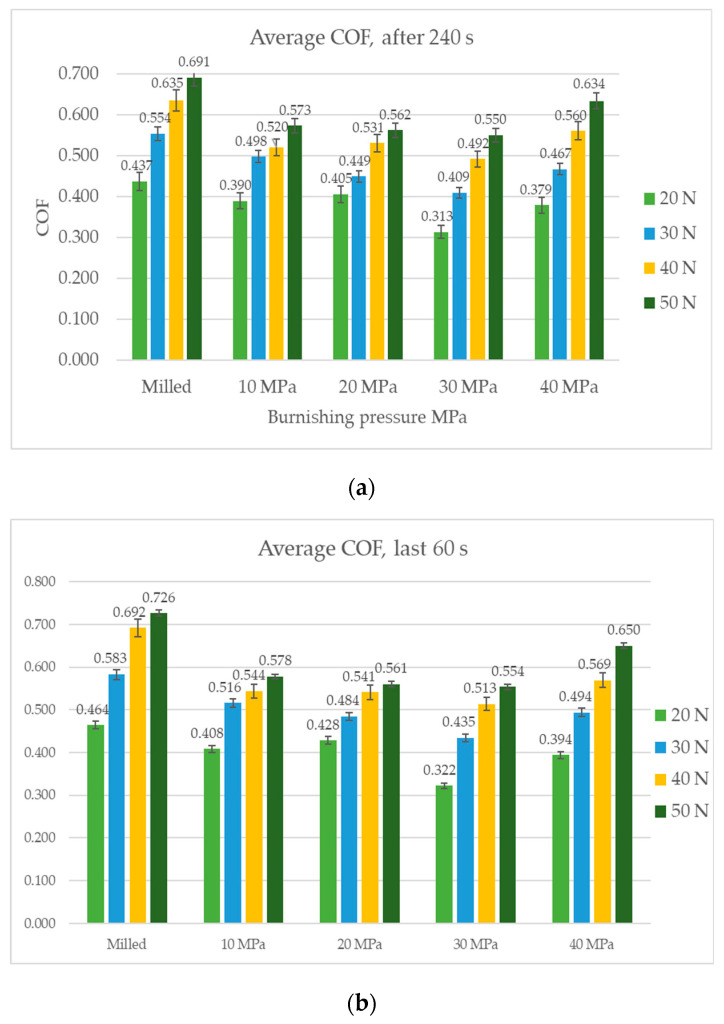
The average coefficient of friction after 240 s (**a**), and for the last 60 s (**b**).

Figure 6 presents the average values of the coefficient of friction with the scatters after its stabilization (after 240 s) and in the final test part (the last 60 s). The finishing values of the friction coefficient were higher than the average values. A growth in the normal force caused an increase in the friction coefficient. The scatters of the average coefficients of friction were typically smaller than those of the final friction coefficients. For the smallest load of 20 N, the differences between the average coefficients of friction for assemblies containing burnished samples with pressures of 10, 20, and 40 MPa were negligible. Burnishing pressure of 30 N caused a decrease in the friction coefficient of 30%. When the normal load was 30 N, ball burnishing with a pressure of 20 MPa led to a smaller friction force in comparison to pressures of 40 MPa and of 10 MPa. Under the normal load of 30 N, burnishing with the pressure of 30 MPa caused a reduction in the friction coefficient of about 45%. When normal loads were 40 and 50 N, from among burnished samples the highest coefficients of friction were obtained for the highest burnishing pressure of 40 MPa. For the normal loads of 40 and 50 N, ball burnishing led to friction reduction of about 20–25%. For the largest normal load, differences between the average coefficients of friction for discs burnished with pressures of 10, 20, and 30 MPa were negligible.

**Figure 7:**
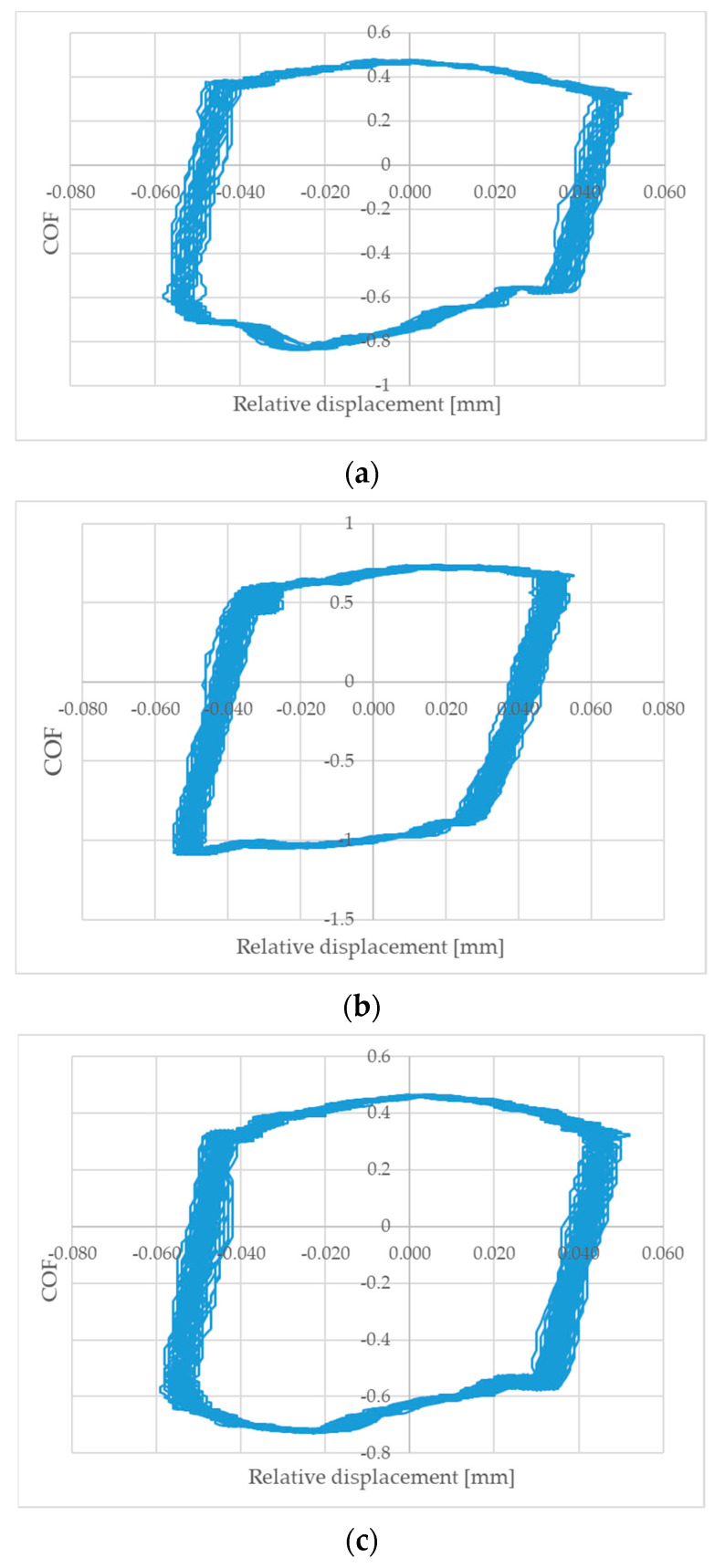
Fretting loops for sliding pairs with discs: (**a**) milled−normal force 50 N, (**b**) burnished−pressure 30 MPa and normal force 20 N, (**c**) burnished−pressure 30 MPa and normal force 50 N.

Figure 7 presents examples of fretting loops obtained after 10 min of tests. The shapes of loops are characteristic of gross slip. The values of the slip index [20,21] were between 5 and 7.

**Figure 8:**
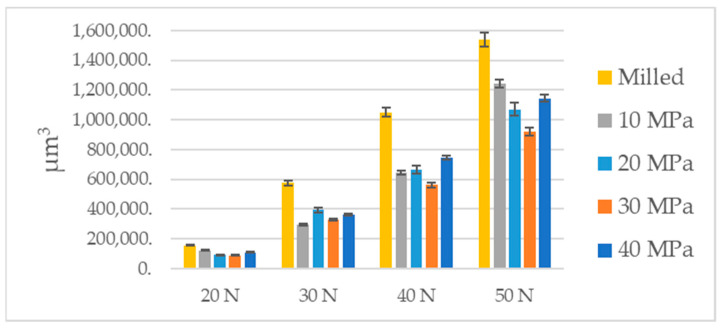
The results of wear examination of disc samples.

Table 2 and Figure 8 present the results of wear examination of elements of the tribological system. Wear levels of discs were higher than those of balls. Typically, the ratio of ball wear to disc wear ranged between 0.2 and 0.3. A growth in the normal load caused a growth in wear volumes of the tribological system. In all analyzed cases ball burnishing led to reduction in wear. For the lowest load, the lowest wear was achieved for burnishing pressures of 20 and 30 MPa. The highest reduction of the volumetric wear was 43%. For the normal load of 30 N, the lowest wear was achieved for burnishing pressure of 10 MPa, followed by 30 MPa, the highest reduction in total volumetric wear due to burnishing was 47%. For the normal load of 40 N, the lowest wear was obtained for the burnishing pressure of 30 MPa, followed by 10, 20, and 40 MPa. The highest reduction in wear volume due to ball burnishing was 47%. When the highest normal load of 50 N was applied, the lowest volumetric wear of the tribologic system was obtained when the burnishing pressure was 30 MPa, followed by 20, 40, and 10 MPa—the largest reduction in wear was 40%. Generally, the smallest wear was obtained for the burnishing pressure of 30 MPa, when the normal loads were 10, 30, and 40 N.

**Figure 9:**
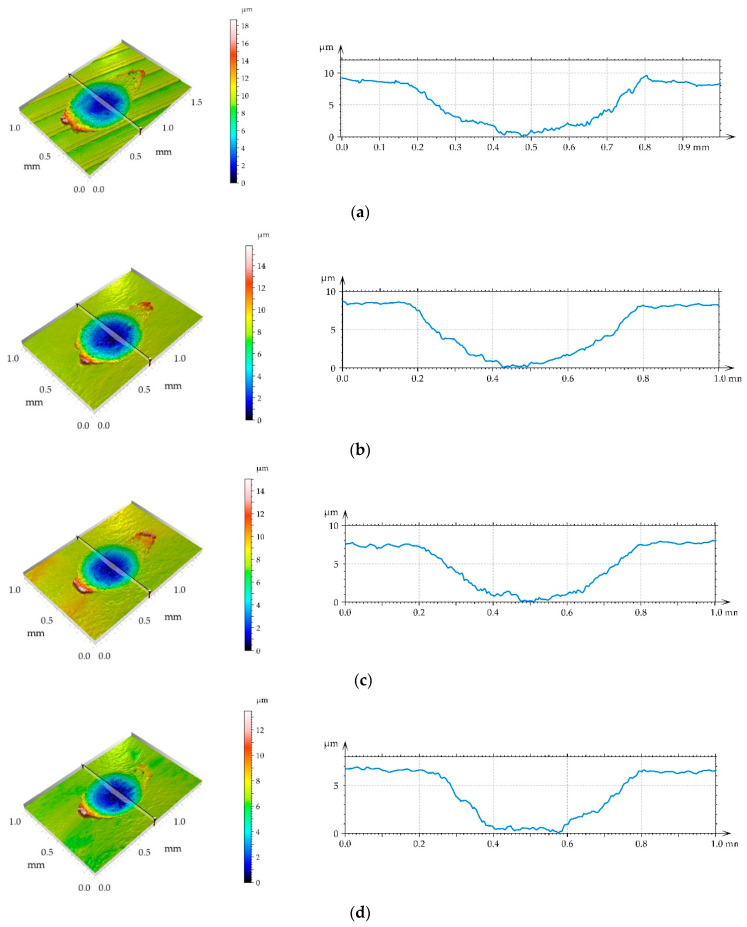

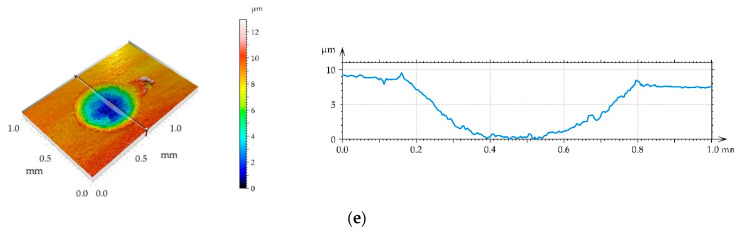
Isometric views of disc surfaces and profiles orthogonal to wear scars at a normal load of 50 N, (**a**) milled sample, (**b**) burnished samples for pressure of 10 MPa, (**c**) 20 MPa, (**d**) 30 MPa, (**e**) 40 MPa.

**Figure 10:**
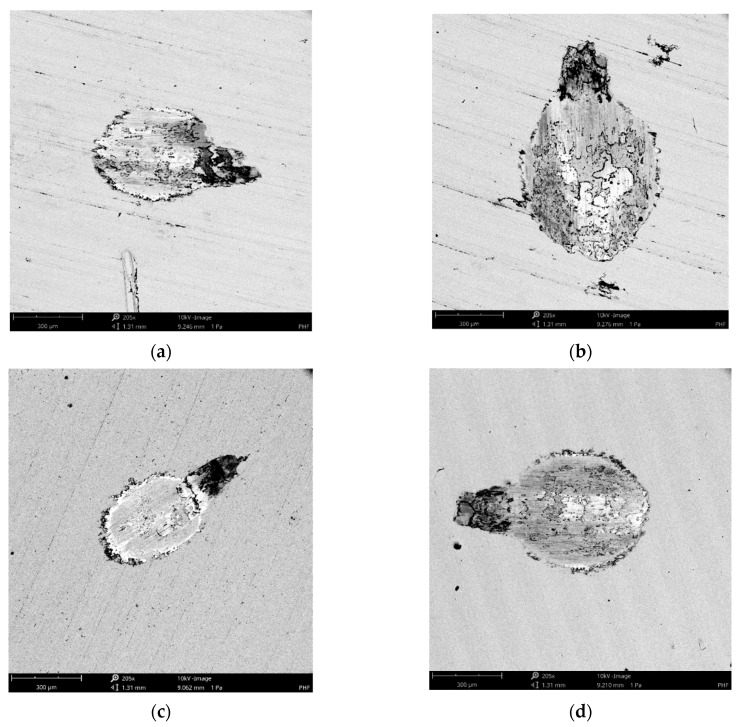
The SEM views of the milled disc surface after tribological tests for the normal load of 20 N (**a**), 40 N (**b**), of burnished disc surface with pressure of 10 MPa for the normal load of 20 N (**c**) and of burnished disc surface with a pressure of 30 MPa for the normal load of 40 N (**d**).

Figure 9 shows views and surface profiles containing wear scars for the highest normal load of 50 N. Figure 10 presents selected SEM images of wear scars on disc samples.

As wear tracks on the disc surfaces had a U shape, abrasion was a dominant type of wear. Plastic deformation of the discs also occurred near the edges of wear scars. Wear was caused by a difference between the values of hardness of two counterparts. As the hardness of the ball was much harder than the hardness of the disc, the wear of the disc was larger than the wear of the ball. Adhesive junctions were not observed, because disc and balls were made of various materials of different hardness.

The smallest coefficients of friction were obtained for ball burnishing with a pressure of 30 MPa, the results were substantial for low normal loads of 20 and 30 N. Similarly, ball burnishing with this pressure led to the smallest wear values of the tribologic system for loads of 10, 30, and 40 N. Therefore, for the burnishing pressure of 30 MPa, the best performance of tribologic system occurred. This behavior was probably related to the smallest roughness height from all analyzed disc samples. The values of parameters Sa, Sq, Sp, Sz and rms. slope were the smallest. There are two sources of friction: deformation and adhesion of contacted summits. For the contact of ceramic ball with steel disc the adhesion effect was negligible, see Figure 9 and Figure 10. Friction due to deformation of asperities decreased when roughness height decreased. Furthermore, the disc sample obtained with burnishing pressure of 30 MPa was characterized by a negative value of skewness Ssk. The beneficial effects of negative skewness on the tribological performance of sliding elements were previously found under lubricated and dry friction regimes [6,36,37,38]. In addition, ball burnishing caused an increase in microhardness of 10%. From among other samples, the highest frictional resistances were obtained for the highest burnishing pressure of 40 MPa, when the highest loads, of 40 and 50 N, were applied. This sample led to the largest volumetric wear of the tribological system for the normal force of 30 N, however, wear levels were also comparatively high when other normal loads were applied. This performance was probably caused by surface texture characterized by comparatively high roughness amplitude and slope (the highest from all burnished discs), for example, the Sq parameter of this sample was about 2.5 times higher than that obtained for the burnishing pressure of 30 MPa. In addition, in contrast to other burnished samples, this surface was not homogeneous.

The highest reduction of the friction force and wear volumes due to ball burnishing was near 45%.

The milled sample of the highest roughness, the highest positive skewness and the smallest microhardness produced the highest friction and wear.

An increase in disc roughness height led to an increase in friction and wear of the analyzed tribological system. Similar effects were obtained in [32]. Wear was proportional to friction. The growth in the unitary pressure caused the increase in friction. This performance was caused by the lack of accommodation of surfaces made of various materials in contact. Wear of ball was smaller than wear of disc. This behavior was caused by very high hardness of balls and low affinity between steel and ceramics. The last property caused a lack of formation of adhesive junctions—abrasion was a dominant wear type.

## 4. Conclusions

- Ball burnishing of steel disc in contact with a ceramic ball under dry gross fretting led to reductions in the resistance to motion and volumetric wear up to 45% in comparison to the milled surface.
- The best tribological performance was achieved for the disc sample created with a burnishing pressure of 30 MPa. This disc was characterized by the smallest roughness height and slope and increased microhardness compared to the milled surface.
- The worst tribological behavior was obtained when the burnishing pressure was 40 MPa. In this case, the non-homogeneous surface had the highest roughness among the burnished discs.
- Wear levels of discs were smaller than those of balls. The wear had an abrasive character with plastic deformation. The growth in normal load led to the increase in the coefficient of friction. Wear of the tribological system was proportional to friction.
- Ball burnishing led to reductions in roughness height and surface slope and to an increase in microhardness compared to the milled sample. The highest reduction in surface amplitude occurred for burnishing pressure of 30 MPa, but the lowest—of 40 MPa. The lowest microhardness increase occurred for the lowest burnishing pressure of 10 MPa.

## Figures and Tables

**Table 1:** Selected areal parameters of disc surfaces.

Disc	Milled	Burnished			
Parameters	-	10 MPa	20 MPa	30 MPa	40 MPa
Sq, µm	0.577	0.135	0.138	0.110	0.269
Ssk	0.312	0.090	0.164	−0.184	0.060
Sku	2.195	3.527	3.094	3.168	4.121
Sp, µm	1.892	0.986	0.696	0.479	0.935
Sv, µm	2.648	0.806	0.549	0.550	1.080
Sz, µm	4.539	1.792	1.245	1.029	2.015
Sa, µm	0.482	0.106	0.110	0.087	0.202
Sal, mm	0.024	0.199	0.051	0.105	0.151
Str	0.014	0.683	0.341	0.665	0.452
Sdq	0.075	0.013	0.013	0.012	0.023

**Table 2:** The values of volumetric wear of disc V_disc_, of balls V_ball,_ and of tribological system V.

Surface Preparation	Load, N	V_disc,_ µm^3^	V_ball,_ µm^3^	V, µm^3^
Milled	20	105.396	50.140	155.536
	30	430.411	144.959	575.370
	40	849.299	200.362	1049.661
	50	1263.317	275.712	1539.029
Burnished, *p* = 10 MPa	20	93.585	29.066	122.650
	30	210.423	86.378	296.801
	40	505.780	139.739	645.518
	50	1001.992	240.320	1242.312
Burnished, *p* = 20 MPa	20	66.295	24.066	90.361
	30	307.287	85.615	392.902
	40	539.396	125.088	664.483
	50	910.172	159.008	1069.180
Burnished, *p* = 30 MPa	20	45.116	42.675	87.791
	30	250.890	77.478	328.368
	40	493.898	69.223	563.120
	50	746.124	174.991	921.114
Burnished, *p* = 40 MPa	20	74.638	35.157	109.794
	30	302.186	59.039	361.225
	40	601.640	144.338	745.977
	50	994.493	149.121	1143.614

## Data Availability

The data presented in this study are available on request from the corresponding author.
